# Incidence of diabetic retinopathy and its predictors among adult patients with diabetes in Ethiopia: a frailty model

**DOI:** 10.3389/fendo.2025.1462210

**Published:** 2025-03-14

**Authors:** Tagese Yakob, Awoke Abraham, Begidu Yakob, Mesfin Manza Jaldo

**Affiliations:** ^1^ School of Public Health, College of Health Science and Medicine, Wolaita Sodo University, Wolaita Sodo, Ethiopia; ^2^ Division of Monitor and Evaluation, Wolaita Zone Health Department, Wolaita Sodo, Ethiopia; ^3^ Department of Biostatic and Epidemiology, School of Public Health, College of Health Science and Medicine, Wachemo University, Hossana, Ethiopia

**Keywords:** diabetic retinopathy, predictors, incidence, diabetes mellitus, Ethiopia

## Abstract

**Background:**

Diabetic retinopathy (DR) is becoming a more widespread public concern worldwide, leading to visual impairments. It has become the leading cause of blindness among working-age adults globally, despite established treatments that can reduce the risk by 60%.

**Objective:**

This study aimed to determine the incidence of diabetic retinopathy and its predictors among adult patients with diabetes in public hospitals in Central and Southern Ethiopia.

**Methods:**

A hospital-based follow-up study was conducted in selected public hospitals in Central and Southern Ethiopia. A total of 376 participants of newly diagnosed adult diabetes were enrolled from 2015-2023 and the follow-up the date was from date of enrolment to the development of events. The data were collected by reviewing their records and entered in Epi-data version 4.6.0.2 and exported to STATA version 14 for analysis. Descriptive statistics of the variables were obtained. The Weibull model with gamma frailty distribution was fitted. Bivariable and multivariable analyses were done, and variables with a p-value less than 0.05 and a corresponding 95% confidence interval in the final model were used. The model of adequacy was checked.

**Results:**

376 adult diabetic patient records were reviewed with the mean baseline age (± standard deviation) of 34.8±10 years. The univariate frailty was statistically significant (Theta=0.236 (0.131, 0.496)). A total of 376 adult patients with diabetes were followed for 682.894 person-years. Overall, an incidence rate of 14.06/100 person-years. Proteinuria (AHR = 2.21: 95% CI: 1.45, 3.57), cardiovascular disease (AHR = 2.23: 95% CI: 1.34, 4.03), and type II DM (AHR = 2.87: 95% CI: 1.30, 6.13) were identified as significant predictors of diabetic retinopathy.

**Conclusion:**

Overall incidence rate of diabetic retinopathy was high. The most effective way to protect our vision from diabetic retinopathy is to manage diabetes effectively and offer support to high-risk individuals with diabetes. Therefore, healthcare professionals and relevant health authorities should target on addressing these factors in their initiatives to prevent diabetic retinopathy in diabetic patients.

## Background

Diabetes is a serious, chronic condition where the body either cannot produce sufficient insulin, produces no insulin at all, or is unable to effectively use the insulin it produces (1). According to the International Diabetes Federation (IDF) 2021, the global prevalence of diabetes among individuals aged 20 to 79 years is 537 million, representing 9.3% of the population in this age group, with 79.4% of cases occurring in low- and middle-income countries (LMICs) (2). This number is expected to rise to 643 million by 2030 and further increase to 782 million by 2045. Additionally, it is estimated that over 6.7 million people aged 20–79 died from diabetes-related causes in 2021 (2, 3).

In low- and middle-income countries are experiencing a large share of the rapid increase in the prevalence of this disease (4). Over the next 20 years, sub-Saharan African (SSA) nations are predicted to see the fastest global increases in the number of patients with diabetes (5). According to estimates, 77% of global DM epidemic burden in the twenty-first century will fall on developing nations like Ethiopia (6). This also plays a part in Africa’s higher early-life morbidity and mortality. In 2017, it was estimated 5.2% of Ethiopians aged 20 to 79 had diabetes, with 2.6 million cases of the disease reported nationwide (2, 7). Patients with diabetes, especially T2DM, are at increased risk of both microvascular and macrovascular disease (8–11). Nearly all patients with T1DM and over 60% of patients with T2DM will develop retinopathy within the first two decades of diagnosis, which is one of the complications of DM (10).

Diabetic retinopathy (DR) is increasingly recognized as a global public health concern and the most common cause of acquired blindness in adults (12). Despite available treatments that can lower the risk by 60% (13, 14), it remains the leading cause of blindness and vision loss among working-age adults worldwide. DR accounts for approximately 2.6% of global blindness (15) and 4.8% of visual impairment (16). In 2020, around 103.12 million people were affected by DR globally, and this number is projected to rise to 160.5 million by 2045, with low- and middle-income countries facing a disproportionately higher burden (17). Studies in Africa revealed that DR has been found with pooled prevalence of 19-29% (18–23). In Ethiopia had a 19.48% of DR (21, 24). Many risk factors have been linked to diabetic DR such as age (24, 25), the length of DM (24, 25), poor medication adherence (26), poor glycemic control (26, 27), obesity (28), and hypertension, which speed up the development of DR in diabetic patients (25, 26, 29, 30). According to the study, hyperglycemia only accounts for 11% of the overall risk of DR; the remaining 89% may be attributable to other possible risk factors (31). The majority of epidemiological studies on DR conducted in Ethiopia and other eastern African countries have so far only estimated using a single facility. Therefore, the objective of this study was to determine the incidence and its predictors of DR among patients with diabetes in public hospitals in Central and Southern Ethiopia.

## Materials and methods

### Study setting, study design, and period

A hospital-based follow-up study design was conducted from June 1/2023 to July 30/2023 among adult patients with diabetes in five randomly selected public hospitals in the central and southern regions of Ethiopia. In both regions, there are 19 zones and three special woredas (districts) in combination with an estimated total population of more than 16.9 million. During the year of this study, there were 52 public hospitals (compressive specialized, referral, general, and primary) in the region (32, 33). The randomly selected hospitals provide general and specialty health care services along with teaching and research activities. It also provides comprehensive diabetes-related services. The diabetic clinic is a former hospital clinic where care and follow-ups are given to patients with all types of diabetes.

### Population

The source population was all adult patients with diabetes who were followed up at chronic disease follow-up units in selected public hospitals in the central and southern regions of Ethiopia from the year January, 2015 - January, 2023. But, those all selected newly diagnosed adult patients with diabetes were the study population. All adult patients with diabetes aged ≥ 18 years who were diagnosed between January, 2015, and January, 2023, were included in the study. However, those whose date of initiation was not recorded, who had gestational DM, or who had DM or retinopathy at the same time of diagnosis were excluded.

#### Sample size calculation and sampling procedure

STATA version 14 used to determine sample size by using the Schoenfeld formula (34) based on the power approach by considering predictors significantly associated with the incidence of adverse drug reactions (ADRs) from previous studies with the hazard ratios for three predictors significantly associated with diabetic retinopathy in a study conducted at the Felege Hiwot Comprehensive Specialized Hospital, Bahir Dar (35), were calculated (**Table 1**). By considering under the following assumptions: Cox proportional hazard model, 95% confidence level, 80% power, 10% withdrawal probability.

**Table 1:** Minimum sample size calculated for predictors significantly associated with adult patients with diabetes in public hospitals in the central and southern regions of Ethiopia.

Variables	AHR	Event	Probability of Event	Sample size
HTN	2.5	38	0.287	376
Proteinuria	4	17	0.287	198
LDL	3.2	24	0.287	257

The Schoenfeld formula [Schoenfeld, 1983 (24)] was used for manual calculation:


$$E=\frac{\left(\frac{Z\alpha }{2}+ZB\right)2}{\text{P}1(1-P1)(\text{ln}\;\text{HR})2}\;\text{and}\;n=\frac{E}{P(E)}$$

Where: n = Total sample size, *HR* is the hazard ratio of selected covariates, P1 is the proportion of subjects in the exposure group, p2= probability of a harm occurring as result of exposure to hazard (1-p1), E = number of events and P (*E*) is the probability of an event from a previous study.

Therefore, the final sample size for this study was 376. Patients who met the inclusion criteria were included in the study. The sampling procedure followed the same approach used in a similar study (36, 37). Initially, five public hospitals were selected by using simple random sampling method. Then, a sampling frame was created in Excel spread sheets using Health Management and Information Systems (HMIS) card numbers/patient’s medical registration number from each randomly selected hospital’s diabetic registration book for those who fulfilled the inclusion criteria were selected in randomly selected hospitals. After that, the calculated sample size was proportionally distributed to each randomly selected hospital. To select study subjects from each of the randomly selected hospitals, a simple random sampling (SRS) technique was used to select 376 records using a computer-generated random number table. Finally, the selected patient’s medical registration numbers were helped to dig out data from the electronic sheet, follow up patient’s folder, and diabetic registration in the chronic follow-up clinic.

#### Operational definition

DR was defined by both direct and indirect ophthalmoscopy assessments performed by physicians confirmed by fundus photography. DR was defined as a microvascular complication of diabetes that was evaluated by clinical examination or indirect ophthalmoscopy by ophthalmologists and classified as present (yes) or absent (no) from the charts based on ophthalmologists’ decisions (38).

#### Time to diabetic retinopathy

The time to diabetic retinopathy was defined as the duration, measured in months, between the diagnosis of diabetes mellitus and the development of diabetic retinopathy (38). Anevent of interest in our study was adult patients with diabetes those who experienced diabetic retinopathy during the follow-up period (38).

### Data collection procedures

The clinical and epidemiological data for this study were gathered from various sources, including diabetes mellitus (DM) intake forms, DM registration logbooks, electronic databases, patient cards, and monthly follow-up charts. Patient intake forms, clinical records, and laboratory results for biomarkers were reviewed using a structured and pretested questionnaire. This questionnaire was developed based on the follow-up charts used in the hospital and various reviewed literature (36–40) published in English. Health Management and Information Systems (HMIS) card numbers or patients' medical registration numbers were used to locate individual patient records or their data in the electronic database. Baseline data were collected from the date patients began regular follow-up treatment and were tracked until the study's conclusion, the occurrence of an event, or censoring during the study period. The data collection was carried out by health workers from the chronic disease follow-up unit, who were selected based on their experience and educational qualifications.

### Data quality control

To maintain data quality, a well-designed and pretested data extraction checklist was utilized. Data collectors received training and detailed explanations regarding the study's objectives and the data collection process. They were also familiarized with the checklist, and strict supervision was provided throughout the data collection phase.

### Data processing and analysis

The collected data were entered into Epi-data version 4.6.0.2 and then exported to STATA version 14 for further analysis. Appropriate data management techniques were employed to ensure that the data were suitable for analysis. Descriptive statistics were conducted to describe the study population. Patients were counted as a censor, if lost to follow-up, if transferred to another health facility before developing retinopathy, who died, or if not develop DR at the end of follow-up. The survival time was calculated in months using the time between the date of diagnosis of diabetes mellitus and the date of the event (DR) or the date of censoring. The outcome of each patient was categorized into censor or event (diabetic retinopathy). The incidence rate with respect to person-time at risk was calculated.

Different survival analysis models were fitted and compared to select best-fitted model. The conventional Cox proportional hazard model may not fit the data well all the time, and may leads to incorrect inferences when all levels of relevant covariates have not been observed or included. The good fitted model was selected for multivariable analysis, by comparing parametric and semi-parametric model. Even though individuals are similar based on the observed variables some individuals are frailer than others, since there are random variables that varies over the population frailty model was carried out to examine predictors of DR. To examine random variables that vary over the population univariate frailty model was carried out.

An associated variable was tested at a 95% CI and summarized by using an adjusted hazard ratio (AHR) and any variable with a P-value less than 0.05 was taken as statistically significant. The adequacy of the model was checked by the Nelson–Aalen cumulative hazard function against the Cox–Snell residual technique (41). The results were presented in a table, figure, and graph.

### Ethics consideration

Ethical approval and a letter of cooperation were obtained from the Institutional Review Board of Wachemo University, College of Medicine and Health Sciences with reference number: wcu/8456/2023 and the hospital were informed about the study objectives through a written letter. Informed consent was waived by the Institutional Review Committee of Wachemo University. Confidentiality was maintained at all levels of the study. The data were stored on a secured password protection system. All procedures were conducted based on the regulations, guidelines, and principles of the Helsinki Declaration.

### Patients and public involvement

Patients and the public were not involved in the design of the study, the conduct of the study or the dissemination of the findings.

## Results

### Socio-demographic characteristics of the respondents

A total of 376 adult diabetic patient records were reviewed with the mean baseline age (± standard deviation) of 34.8 ± 10 years. Of the total study participants, half of 191 (50.6%) were females, and about 199 (52.9%) were married. About one-third 210 (55.9%) of patients had a college education or above, and about 90 (23.9%) of patients were Government employed. Of the total study participants, greater than one-half, 102 (27.1%), were follow-up in Wolaita Sodo compressive specialized hospital and about 281 (74.7%) were from urban residents (**Table 2**).

**Table 2:** Socio-demographic characteristics of the respondents with patients with diabetes at follow-up at public hospitals in the central and southern regions of Ethiopia.

Variables	Category	Frequency	Percent (%)
Sex	Male	186	49.4
	Female	191	50.6
Marital status	Single	76	20.2
	Married	199	52.9
	Divorced	78	20.7
	Widowed	23	56.2
Residents	Urban	281	74.7
	Rural	95	25.3
Educational status	No formal education	29	7.7
	Primary	29	7.7
	Secondary	108	28.7
	College and above	210	55.9
Occupation	Gov’t employed	90	23.9
	Non-gov’t employed	63	16.8
	Farmer	79	4.5
	Student	32	8.5
	Housewife	63	16.8
	Daily labourers	32	8.5
Follow- up Hospitals*	WSUCSH	102	27.1
	JGH	59	15.7
	NEMMCSH	94	25.0
	DGH	65	17.3
	SGH	56	14.9

*WSUCSH, WolaitaSodo University Compressive Specialized Hospitals; JGH, Jinka General Hospital; NEMMCSH, Nigist Eleni Mohamed Specialized Hospital; DGH, Durame General Hospitals; SGH, Sawula General Hospitals.

### Behavioral and clinical characteristics

The median duration of DM treatment of patients was 20.2 months, with IQRs of 18.4 and 29.3 months. Three-fourths (n=282, 75.2%) of the patients were receiving insulin treatment, and approximately 73% of patients had good adherence to treatment. In this study, while 17.9% of patients had a history of smoking tobacco products, nearly 12.5% of patients had a history of alcoholic drinks. Among the study subjects, one-fourth (96, 25.5%) of patients had developed retinopathy (**Table 3**).

**Table 3:** Baseline clinical and behavioral characteristics of patients with diabetes on follow-up in public hospitals in the central and southern regions of Ethiopia.

Variables	Category	Frequency	Percent (%)
Body mass index	Underweight	56	14.9
	Normal	303	80.6
	Obesity	17	4.5
Proteinuria	Negative	26	6.9
	Positive	350	93.1
DM treatment types	Insulin	282	75.2
	Noninsulin	74	19.7
	Mixed	19	5.1
Cardiovascular disease	Yes	51	13.6
	No	325	86.4
Adherence	Good	274	73.8
	Fair	72	19.4
	Poor	25	6.7
Retinopathy	Yes	96	25.5
	No	280	74.5
Past opportunistic infections	Yes	84	22.3
	No	292	77.7
DM type	Type I	117	29.8
	Type II	249	70.2
Family History of DM	Yes	138	37.2
	No	227	62.7
Alcohol	Yes	27	7.2
	No	349	92.8
Smoking	Yes	67	17.9
	No	307	82.1
Exercise	Yes	55	14.9
	No	315	85.1

### Incidence of diabetic retinopathy

The patients were followed for a minimum of 2.8 months and a maximum of 59.3 months, with a median follow-up time of 19.3 months and an IQR of 16.6to 26.9. Out of 376 study participants who were followed during the investigation,231 (61.43%) were alive and continued their treatment in the health facilities, 11 (2.92%) were lost follow up, 29(5.05%) were transferred to other facility, 9 (2.39%) were died, and 96 (26.5%) developed retinopathy. The incidence rate was 14.06/100 person-years (PY) (approximately fourteen cases per 100 PY of observation), with a 95% CI of (9.82-17.61).

### Survival probability of patients with diabetic and proportional hazard assumption

**Figure 1:**
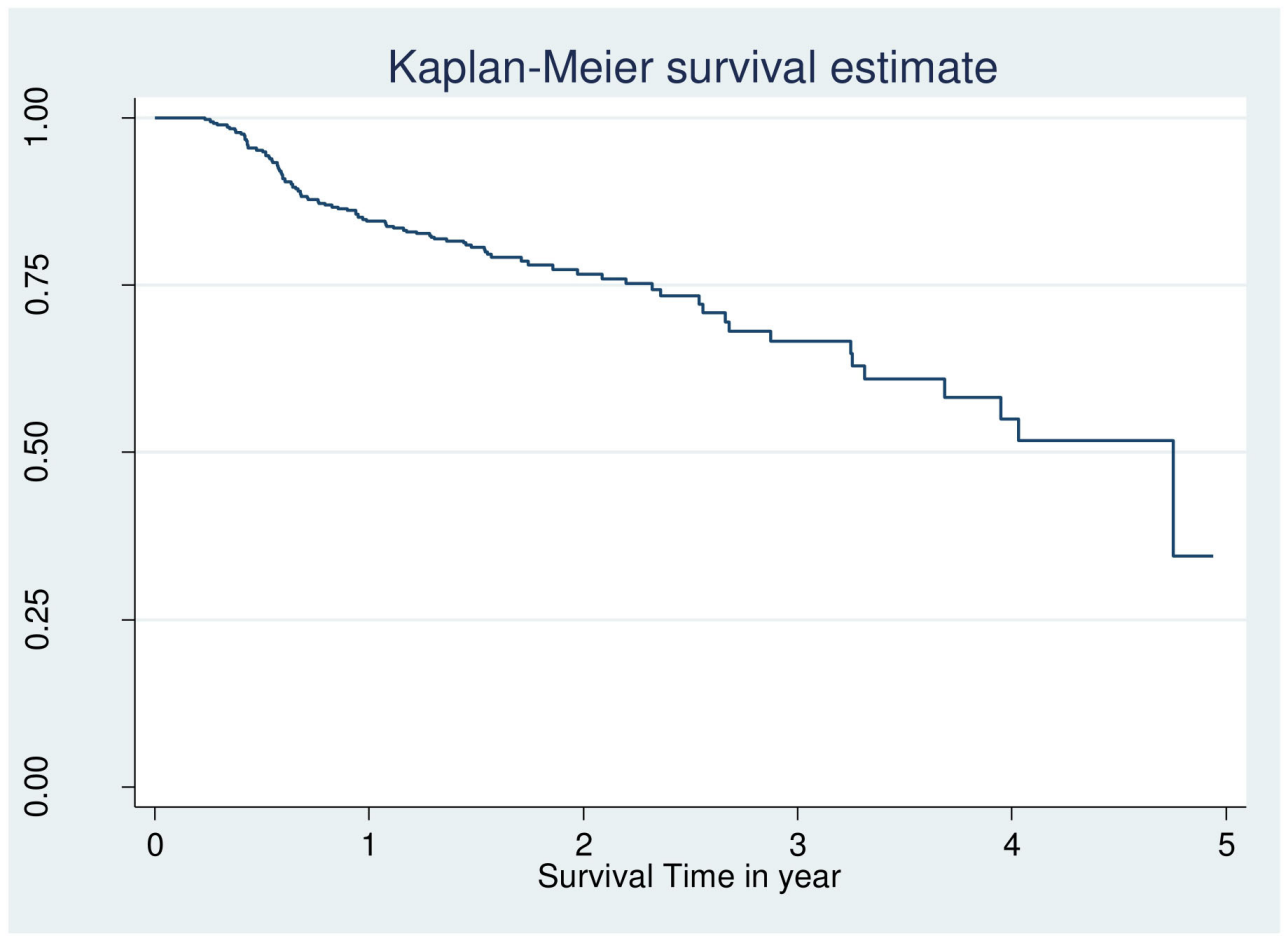
Overall Kaplan Meier failure estimate of retinopathy among patients with DM at public hospital in central and southern, Ethiopia, 2023.

**Figure 2:**
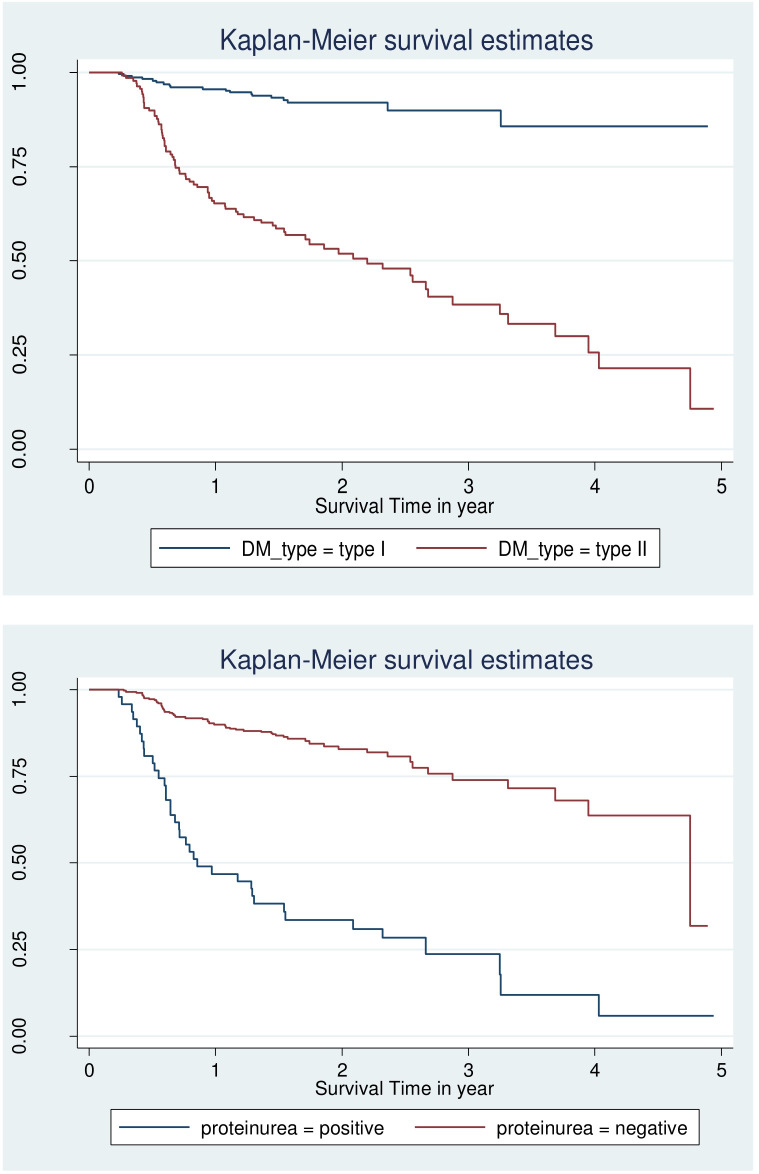
Log-log survival plot by DM type and protein uria for patients with DM at selected public hospital in central and southern, Ethiopia, 2023.

However looking at the Kaplan–Meier survival for CVD, adherence, body mass index is not enough to be certain of proportionality even though few variable fulfil criteria and overall KM go down (**Figures 1**, **2**).

Different techniques of survival analysis were tried to find a model that best fits the data. First, the proportional hazard model was fitted; then, the Weibull, exponential, log-logistic, Gompertz and log-normal parametric survival models were fitted. Finally, these parametric models with the gamma and inverse Gaussian univariate frailty models were fitted. Out of which the one that gave the best fit was selected based on AIC and BIC. Schoenfeld residual test for the Global test was insignificant indicating the proportional hazard assumption holds (**Table 4**).

**Table 4:** Schoenfeld residual test to check proportional hazard assumptions.

Variables	Chi-square	DF	p-value
Sex	0.1378	1	0.519
Adherence	2.1380	2	0.298
DM duration	0.4501	1	0.497
Body mass index	2.1219	2	0.201
Smoking	0.4132	1	0.397
Past OI	5.6836	1	0.137
Alcohol	0.0342	1	0.707
DM type	1.7109	1	0.131
DM treatment	0.2508	1	0.791
Family history of DM	9.7817	1	0.079
CVD	0.5350	1	0.298
Proteinuria	3.0085	1	0.215
Global	22.1680	15	0.375

#### Predictors of the incidence of diabetic retinopathy among diabetic patients

Based on the p-value of the Bivariable Cox proportional hazard regression analysis, twelve variables with p-value ≤ 0.20 were identified as potential candidate variables for the multivariable Cox proportional hazard regression model. These were sex, educational status, BMI, CVD, protienuria, adherence, family history of DM, DM type, past opportunistic infections, smoking, and alcohol. In multivariable cox proportional hazard regression analysis, CVD, DM type, and protienuria showed statistically significant associations with the incidence of Diabetic retinopathy (**Table 5**).

**Table 5:** Bivariate and multivariable cox proportional hazard regression analysis results for predictors affecting diabetic retinopathy in selected public hospitals of Central and South region of Ethiopia.

Variables	Category	Censored (%)	Event (%)	CHR (95%CI)	AHR (95%CI)	p-value
Sex	Male	144 (77.4)	42 (22.6)	1	1	
	Female	137 (71.7)	54 (28.3)	1.25 (0.90- 2.02)	2.72 (0.78 - 3.74)	0.095
Educational status	No formal education	13 (44.8)	16 (55.2)	0.97 (0.54- 1.74)	0.93 (0.67 - 1.90)	0.167
	Primary	17 (58.6)	12 (41.4)	1.81 (0.98- 3.31)	0.46 (0.25 - 2.65)	0.071
	Secondary	77 (71.3)	31 (28.7)	2.24 (0.95- 5.24)	4.09 (0.56 - 4.72)	0.259
	College and above	173 (82.3)	37 (17.6)	1	1	
Body mass index	Underweight	25 (44.6)	31 (55.4)	6.92 (4.39-10.9)	3.40 (0.93 - 3.43)	0.071
	Normal	240 (79.2)	63 (20.8)	8.60 (4.74- 15.6)	2.09 (0.53 – 6.92)	0.167
	Obesity	15 (88.2)	2 (11.8)	1	1	
Proteinuria	Negative	10 (21.3)	37 (78.7)	1	1	
	Positive	270 (82.1)	59 (17.9)	5.98 (3.95- 9.07)	2.21 (1.45, 3.57)*	0.001
CVD	No	265(89.2)	32 (10.7)	1	1	
	Yes	15 (15.6)	64 (84.4)	1.69 (1.13 - 2.54)	2.23 (1.34, 4.03)*	0.002
Past opportunistic infections	No	37 (38.5)	47 (61.5)	1	1	
	Yes	255 (87.3)	49 (12.7)	2.09 (1.37- 3.57)	1.21 (0.49 - 2.43)	0.120
Family history of DM	No	45 (38.7)	72 (61.5)	1	1	
	Yes	227 (91.2)	22 (8.8)	4.30 (5.19- 13.5)	1.38 (0.60 - 3.32)	0.095
DM type	Type I	208 (91.6)	19 (8.4)	1	1	
	Type II	61 (44.2)	77 (55.8)	6.64 (4.62- 12.7)	2.89 (1.21-7.18)*	0.035
Alcohol	No	11 (44.0)	16 (56.0)	1	1	
	Yes	269 (77.1)	80 (22.9)	5.04 (3.91- 8.84)	1.28 (0.66 - 2.69)	0.087
Smoking	Yes	261 (85.0)	46 (15.0)	1	1	
	No	17 (25.4)	50 (74.6)	5.19 (3.46- 7.79)	0.81 (0.40 - 1.56)	0.075
	P			1.395 (1.147, 1.605) *		
	Theta			0.236 (0.131, 0.496) *		

P is the shape parameter of Weibull distribution; Theta is the parameter for the frailty; CI, confidence interval; DM, diabetic mellitus; AHR, adjusted hazard ratio; CHR, crude hazard ration, *p < 0.05.

#### Model adequacy

**Figure 3:**
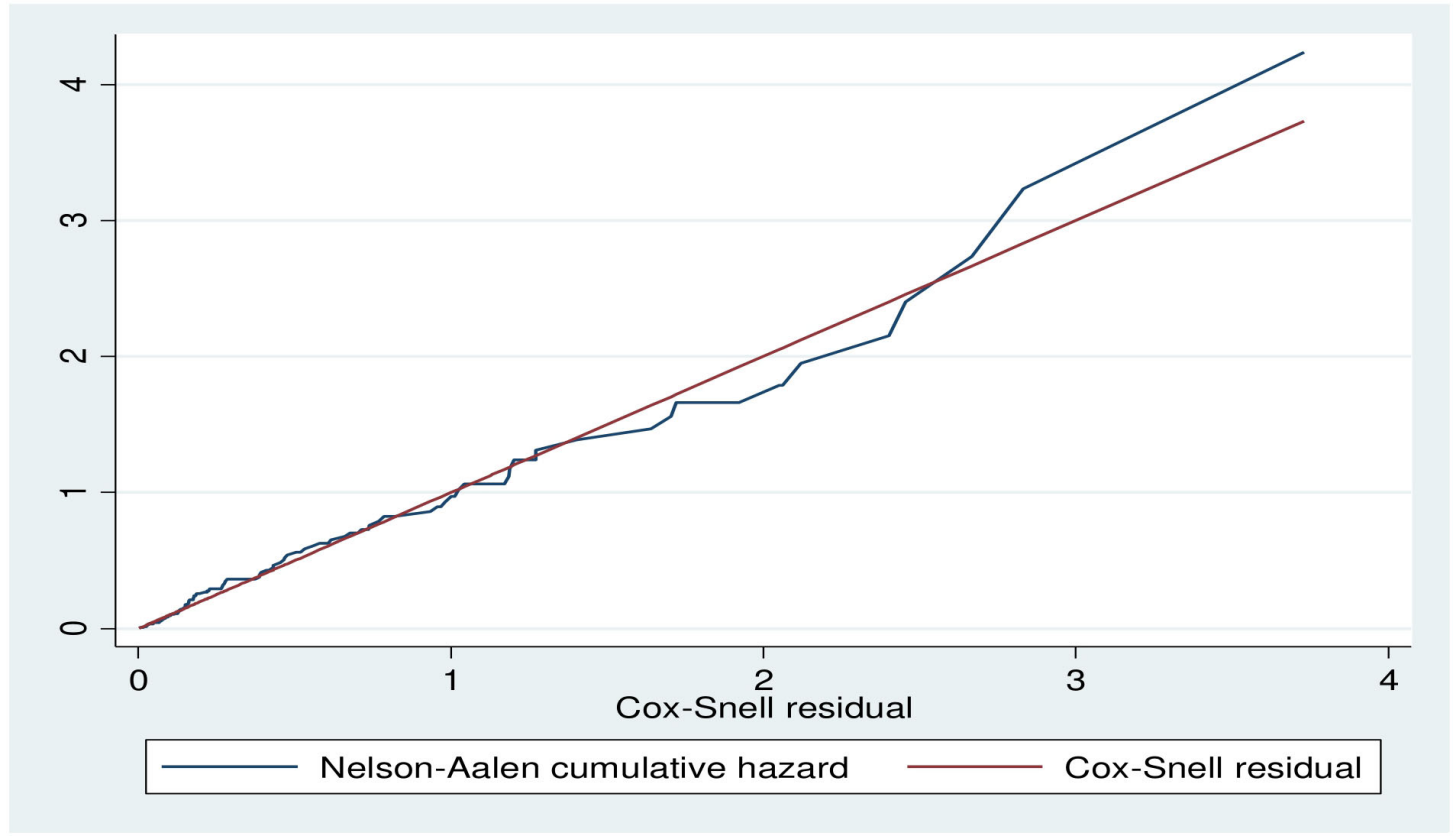
Cox-Snell residual plot of Diabetic retinopathy among DM patient public hospital in central and southern, Ethiopia, 2023.

The Cox-Snell residual plot is approximately linear through the origin with a slope of 1 which indicates that the fitted Cox model is adequate (**Figure 3**).

## Discussion

This study sought to ascertain the incidence of diabetic retinopathy and its risk factors among patients with diabetes in the central and southern regions of Ethiopia. According to this study, the cumulative density of diabetic retinopathy was 14.06/100 person-years (PY) with a 95% CI of (9.82-17.61). This finding was comparable with those of studies conducted in Addis Ababa, Ethiopia (32), in ArbamichGeneral Hospital, Ethiopia (40), in Jimma Medical Center, Ethiopia (39), in Japan (42), in Spain (43) and in United Kingdom (44). This might be due to the use of a matching service delivery approach at the diabetic clinic in the facilities.

The current study revealed that the incidence was higher than that of studies conducted in China (45), Spain (46), the United States (47), and Australia (48). This discrepancy may be the result of variation in the study participants’, demographics, early diagnosis, and follow-up care for diabetic patients. Additionally, the incidence of DR may be reduced in in China, Spain, the United States, and Australia by having strict monitoring of complications and high-quality healthcare systems (47).

The results of this study, however, were lower than those of studies carried out in Bangladesh (49), England (50), Kenya (51),or South Africa, where a retrospective follow-up analysis revealed a cumulative incidence of DR 47/1000 people over the course of a 7 years follow-up study (52).This difference might be due to the study period and study population used in the respective studies, as the follow-up years and screening programs could all contribute factors.

This study identified proteinuria as a risk factor for DR. The hazard of DR was 2.21 times greater than among patients with positive proteinuria than those with negative proteinuria. This finding is in line with those of retrospective cohort studies conducted in the Felege Hiwot Comprehensive Specialized Hospital, Ethiopia (35) and Iran (53). On the other hand, a study revealed that, there was no difference between the presence and absence of DR in terms of the albumin excretion rate (AER) (35). This may be connected to retinal pathologic features linked to inflammatory processes in kidney infection. These processes include the thickening of the retinal basement membrane, which causes DR, circulatory irregularities, and decreased vascular responsiveness (54). Furthermore, this can be a result of the different study designs and study focuses.

CVD has been identified as another risk factor for the incidence of DR among adult diabetic patients. The hazard of DR among patients with diabetes with CVD comorbidities was 2.23 times greater than that among patients with diabetes with no CVD. This finding is consistent with those of studies conducted in China (45), Japan (42), Taiwan (55), Dessie, Ethiopia (56), Southwest Ethiopia (39), in Northwest Ethiopia (35), and ArbamichGeneral Hospital, Ethiopia (40). This finding is also analogous to those of studies conducted in Hong Kong (57) and Denmark (58). This could be the result of chronic hyperglycemia in the as of hypertension activating the renin-angiotensin system, which led to an increase in the level of Angiotensin II (AII) in vitreous fluid in patients with diabetics macular edema and DR. Drastic reduction in blood flow is ultimately caused by enhanced vascular permeability and neovascularization (59). Also, this significant link between the outcome variable and hypertension might be due to the repeated clinical coexistence of hypertension and DM (57). Furthermore, hypertension itself might cause complications of DM, such as DR, through changes in the morphology of the vessel at the retina, such as hemorrhages, hard exudates, and others (60).

In this study, an expected hazard ratio of DR was 2.89 (95%CI 1.21–7.18) higher in patients with T2DM than patients with T1DM while keeping other covariates keeping constants. These findings are consistent with a study performed at Ayder Referral Hospital in Ethiopia (61) and in Addis Ababa, Ethiopia (32), which revealed that T2DM were more likely than T1DM patients to experience microvascular problems earlier in life. This difference may be attributed to the fact that aging is more prevalent among Type 2 Diabetes Mellitus (T2DM) patients compared to Type 1 Diabetes Mellitus (T1DM) patients. Furthermore, the onset of T2DM tends to decrease with age, and microvascular diabetic complications can develop over similar durations in both types (62). In contrast to our findings, studies conducted in Scotland (43) and Spain (63) reported that T1DM patients were more likely to develop complications than T2DM patients. These discrepancies could be due to differences in the study populations. For instance, the Spanish study included all participants with T1DM and T2DM who were 2 years or older at the start of the study (not at the time of diagnosis), whereas our study only included patients who were 18 years or older at diagnosis. This may have excluded a significant portion of T1DM patients, potentially influencing the results.

Even though the current study had some strengthen such there are some drawbacks of the current study such as; as small sample used and a result of the study focusing on hospital patients, it does not accurately represent the incidence of DR in the general diabetic population as undiagnosed subjects may have been excluded so that more randomized and stratified sampling method and large sample size across different healthcare facilities with extending the follow-up period with prospective study design further required. Because the study was retrospective, we missed some important diabetes complications that had a significant association with DR, such as lipid profile, self-monitoring of blood glucose practice, hemoglobin level, presence/absence of other diabetic complications, and residents, because we obtained the data through chart review.

## Conclusion

The overall incidence of retinopathy among patients with diabetes was 14.06/100 person-years observations. For this study, the predicted median follow-up time was 57 months. CVD, proteinuria, and diabetes type were identified as predictors of DR. To reduce diabetic retinopathy the best strategy to protect our eyesight from diabetic retinopathy is to keep our diabetes under control and provide high-risk individuals with diabetes. Health professionals and relevant authorities should focus on diabetic patients who also have CVD and proteinuria as part of their efforts to reduce the risk of diabetic retinopathy. Regular monitoring, evaluation, and documentation of these factors are essential. Healthcare providers in diabetes follow-up clinics should work to enhance patients' self-care practices and overall quality of life to decrease the incidence of diabetic retinopathy. Additionally, they should ensure that all diabetic patients receive the WHO-recommended eye evaluation at least twice a year. While follow-up frequency may vary depending on blood glucose control, it is advisable for patients to undergo a general assessment during each follow-up, particularly focusing on ocular health.

## Data Availability

The raw data supporting the conclusions of this article will be made available by the authors, without undue reservation.
